# The learning effect of using stereoscopic vision in the early phase of laparoscopic surgical training for novices

**DOI:** 10.1007/s00464-017-5654-2

**Published:** 2017-06-22

**Authors:** Hitoshi Harada, Shingo Kanaji, Masayasu Nishi, Yoshito Otake, Hiroshi Hasegawa, Masashi Yamamoto, Yoshiko Matsuda, Kimihiro Yamashita, Takeru Matsuda, Taro Oshikiri, Yasuo Sumi, Tetsu Nakamura, Satoshi Suzuki, Yoshinobu Sato, Yoshihiro Kakeji

**Affiliations:** 1 Division of Gastrointestinal Surgery, Department of Surgery, Graduate School of Medicine, Kobe University, 7-5-2, Kusunoki-cho, Chuo-ku, Kobe, 650-0017 Hyogo Japan; 2 Graduate School of Information Science, Nara Institute of Science and Technology, Ikoma, Japan

**Keywords:** 3D laparoscopy, 2D laparoscopy, Novice, Task performance, Training, Learning effect

## Abstract

**Background:**

Recently to improve depth perception, the performance of three-dimensional (3D) laparoscopic surgeries has increased. However, the effects of laparoscopic training using 3D are still unclear. This study aimed to clarify the effects of using a 3D monitor among novices in the early phase of training.

**Methods:**

Participants were 40 novices who had never performed laparoscopic surgery (20 medical students and 20 junior residents). Three laparoscopic phantom tasks (task 1: touching markers on a flat disk with a rod; task 2: straight rod transfer through a single loop; and task 3: curved rod transfer through two loops) in the training box were performed ten times, respectively. Performances were recorded by an optical position tracker. The participants were randomly divided into two groups: one group performed each task five times initially under a 2D system (2D start group), and the other group performed each task five times under a 3D system (3D start group). Both groups then performed the same task five times. After the trial, we evaluated the performance scores (operative time, path length of forceps, and technical errors) and the learning curves for both groups.

**Results:**

Scores for all tasks performed under the 3D system were significantly better than scores for tasks using the 2D system. Scores for each task in the 2D start group improved after switching to the 3D system. However, scores for each task in the 3D start group were worse after switching to the 2D system, especially scores related to technical errors.

**Conclusions:**

The stereoscopic vision improved laparoscopic surgical techniques of novices from the early phase of training. However, the performance of novices trained only by 3D worsened by changing to the 2D environment.

Laparoscopic surgery has advantages in terms of low invasiveness or esthetic outcome. However, complicated surgery using the laparoscopic approach is still challenging compared with open surgery due to technical limitations with the planar image from two-dimensional (2D) monitors. The recent utilization of stereoscopic vision from a three-dimensional (3D) monitor in laparoscopic surgery has overcome a lack in depth perception and improved surgical performance, such as operative time and accuracy [1–7]. Further, laparoscopic performance using a 3D monitor by novices who had never performed laparoscopic surgery was also improved in relation to superior accuracy and dexterity compared with that under 2D [8–12]. However, the negative or positive effects of laparoscopic training of novices using 3D vision are still unclear. In this study, we compared operative data, including the path length of forceps, under 3D and 2D vision by 3D optical tracking systems in a laparoscopic training box and clarified the effects of 3D vision for novices in the early phase of training.

## Materials and methods

### Training equipment

**Fig. 1:**
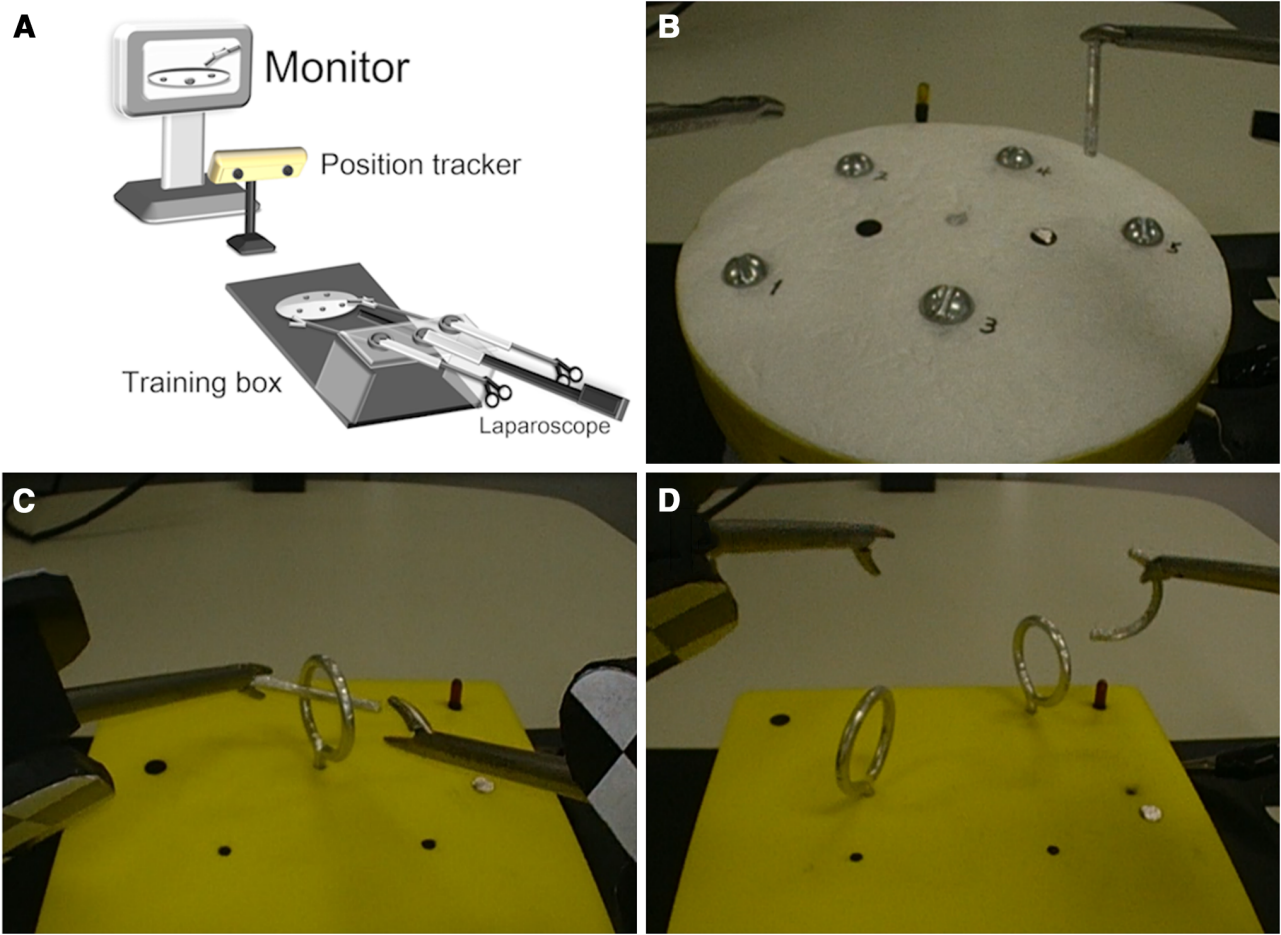
Setup for recording performance of participants. **A** The apparatus consisted of a monitor, position tracker, and training box including a phantom task. **B** Device for task 1 (touching markers on the flat disk with a rod). **C** Device for task 2 (straight rod transfer through single loop). **D** Device for task 3 (curved rod transfer through two loops)

Figure 1A shows the training setup. For the assessment of laparoscopic performance using stereoscopic images captured by a 3D system, we set the monitor on place where a subject directly faced the display with viewing distance ranged about 1.2 m and viewer’s eye level is the same as the middle of the display. This setting was simulated as similar as possible to the usual position of surgeon and monitor in actual laparoscopic surgery in our institution. The distance between the tip of the endoscope and the object of the operation was fixed at 15 cm.

Devices used were a 3D laparoscope and 3D laparoscopy system (Olympus Medical Systems, Tokyo, Japan). With this laparoscope, two charge-coupled device (CCD) image sensors are located at the distal end of the laparoscope to provide left and right images, respectively. These two image signals are processed by a special-purpose video system to generate a high-resolution 3D image that is then displayed on a 3D monitor and viewed through 3D glasses to provide realistic 3D images. This system can be switched from providing 3D images to 2D images by pressing a switch on the scope.

The micron tracker (Claron Technology, Toronto, Canada) consists of CCD image sensors. This system enables the tracking and recording of 3D coordinates and rotation of forceps with an optical marker. Data are obtained as text data and can be analyzed by statistical software.

### Study design

**Fig. 2:**
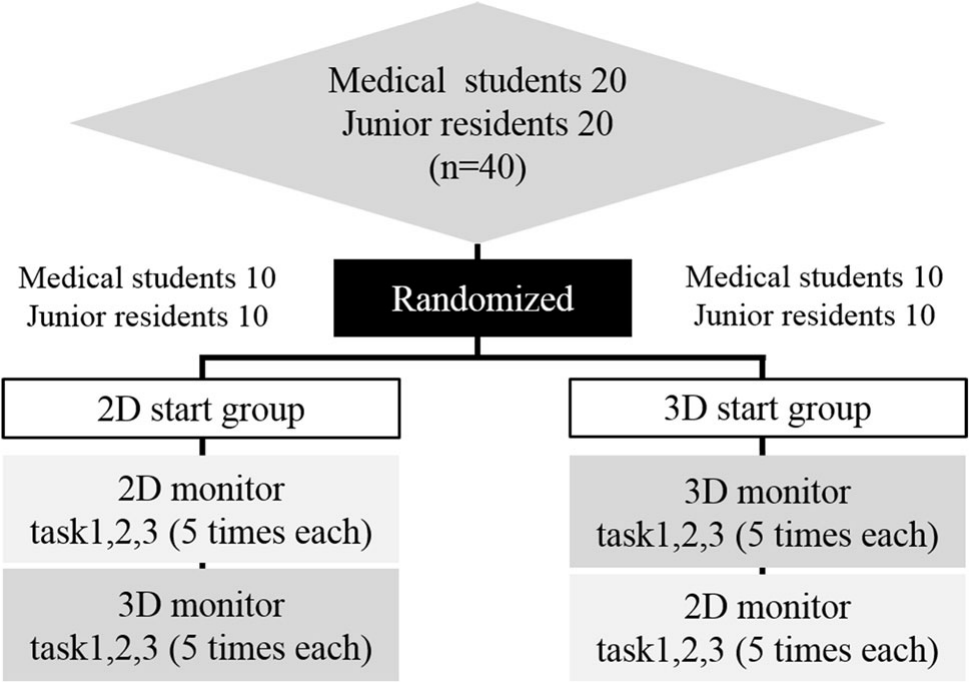
Diagram of study design

Study participants were 40 novices who had never performed laparoscopic surgery as an operator (20 medical student volunteers and 20 junior resident volunteers). The sample size was determined based on the outcome of our preliminary study. Participants performed three different laparoscopic phantom tasks in the training box five times each (one set) and performances were recorded by the optical position tracker in order to analyze the motion of forceps in a 3D space. Participants were randomly divided into two groups: one group performed a set of tasks initially under the 2D system (2D start group) and the other group performed the tasks under the 3D system (3D start group). Then both groups again performed the same set of tasks after switching to the alternate system (Fig. 2). All participants completed the tasks using 2D and 3D monitors with the same settings and on the same day. After participants completed the second set of tasks, we compared operative times, path lengths of forceps, and technical errors between systems. In addition, we investigated the learning curves for each group throughout all attempts.

The purpose of the study was sufficiently explained, and consent was obtained from all participants of this study before the trial.

### Tasks

#### Task 1: touching markers on the flat disk with a rod (a simple operation) (Fig. 1B)

The metal rod on the center of the flat disk was grasped with a lockable, endoscopic needle holder by the right hand, then was moved to touch the metal markers in order from No. 1 to 5. After touching the No. 5 marker, the rod was passed to a needle holder in the left hand and then moved back to touch the metal markers No. 4–1 in that order. Failure to pass the rod from the right to the left hand and not touching the markers were counted as errors.

#### Task 2: straight rod transfer through single loop (Fig. 1C)

The metal rod on the right side of the flat table was grasped with an endoscopic needle holder by the right hand and then was transferred through the loop on the center of the table, without touching the loop, to the left side. After the transfer, the rod was passed to a needle holder in the left hand and touched the black dot on the left side. The rod was then moved back through the loop to the right side and passed back to the right hand. Finally, the rod touched the black dot on the right side. Accidental touches between the rod and loop were counted as errors.

#### Task 3: curved rod transfer through two loops (Fig. 1D)

The curved metal rod on the right of the flat table was grasped with an endoscopic needle holder by the right hand and then was transferred through the loop on the center of the table, without touching the loop, to the left side. After its transfer to the left side, the rod was passed to a needle holder in the left hand and touched the black dot on the left side. The rod was then moved back through the other loop to the right side and was passed back to the right hand. Finally, the rod touched the black dot on the right side. Accidental touches between the rod and loop were counted as errors.

### Statistical analysis

Measured data were recorded and assessed by a single investigator. Forceps path lengths were calculated as follows:

$$P_{1} \left( {x_{1} , \, y_{1} , \, z_{1} } \right), \, P_{2} \left( {x_{2} , \, y_{2} , \, z_{2} } \right), \, \ldots \, P_{n} \left( {x_{n} , \, y_{n} , \, z_{n} } \right)$$

$${\text{Sum of path lengths}} = \sum\limits_{n = 2}^{N} {\sqrt {(x_{n} - x_{n - 1} )^{2} + (y_{n} - y_{n - 1} )^{2} + (z_{n} - z_{n - 1} )^{2} } },$$

where *P*_1_, *P*_2_, … *P*_*n*_ represent tip position of the forceps at each time frame (one time frame = 0.05 s).

Statistical analysis of the data was performed using JMP ver.8.0 software (SAS Institute Inc., Cary, NC, USA). All data are presented as the median value and the mean ± standard error of the mean (SEM). The comparisons between groups were performed by the Wilcoxon signed-rank test or the Wilcoxon rank-sum test. *P* < 0.05 was considered significant.

## Results

### 3D monitor versus 2D monitor

**Fig. 3:**
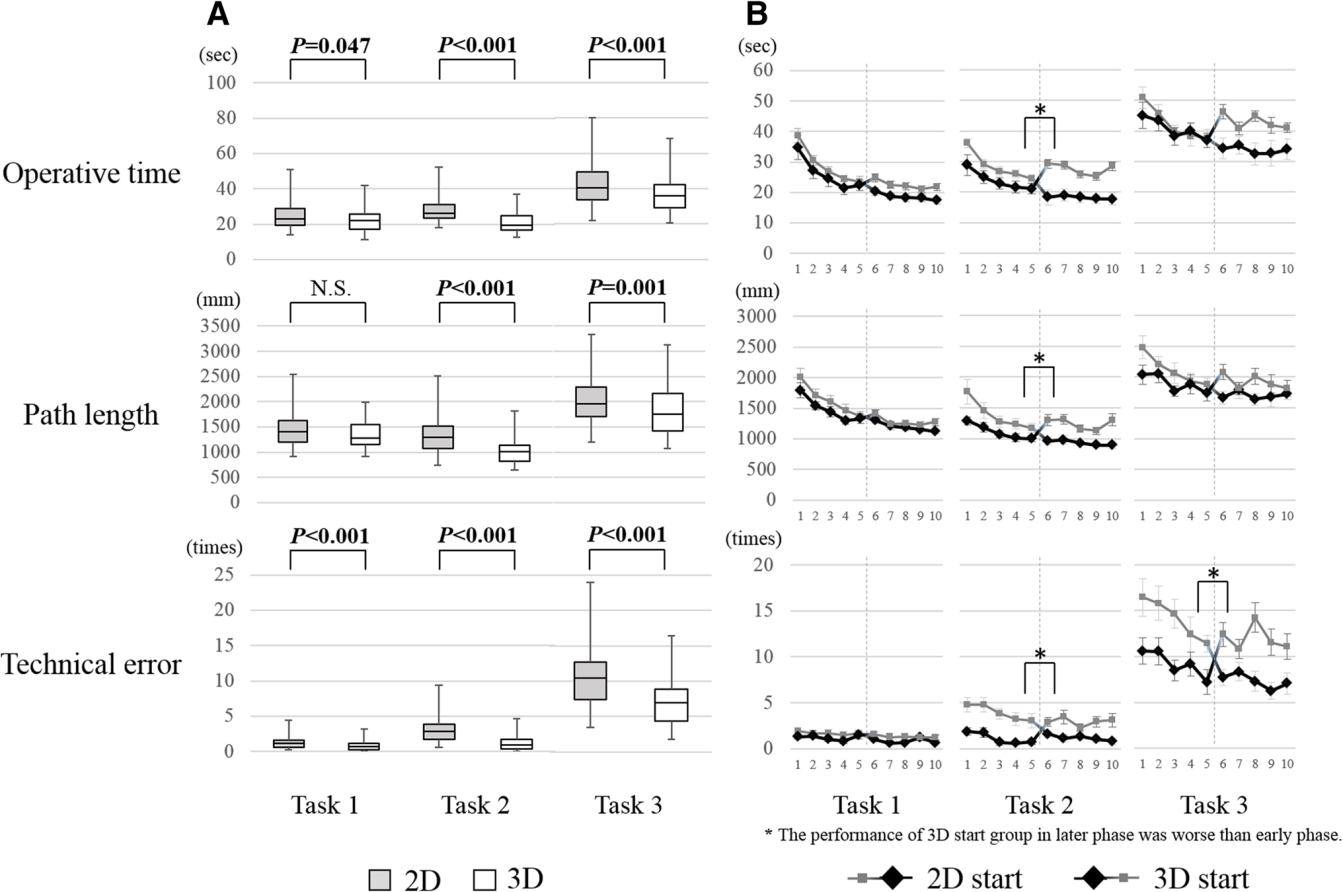
**A** Comparison between 2D and 3D systems regarding average scores (operative times, path length of forceps, technical errors) of all participants. **B** Learning curves of 2D start group and 3D start group. During early phase of training, the performance of the 3D start group was superior to the 2D start group. However, during the later phase of training, the performance of the 3D start group became worse than the 2D start group

Figure 3A shows comparisons of the average scores of all participants for each task between 2D and 3D systems. Median operative times were 22.9 and 22.0 s (*P* = 0.047) for task 1 with 2D and 3D systems, 26.2 and 19.5 s (*P* < 0.001) for task 2 with 2D and 3D systems, and 40.4 and 36.0 s (*P* < 0.001) for task 3 with 2D and 3D systems, respectively. Median path lengths were 1407.3 and 1276.6 mm (*P* = 0.120) with 2D and 3D systems for task 1; 1291.1 and 1009.1 mm (*P* < 0.001) with 2D and 3D systems for task 2; and 1961.7 and 1759.5 mm (*P* = 0.001) with 2D and 3D systems for task 3, respectively. Median technical errors (times) were 1.2 and 0.7 (*P* < 0.001) with 2D and 3D systems for task 1; 2.8 and 0.9 (*P* < 0.001) with 2D and 3D systems for task 2; and 10.5 and 6.9 (*P* < 0.001) with 2D and 3D systems for task 3, respectively. Each score for all tasks performed under 3D was significantly better compared to those with the 2D system except for mean path length in task 1. Although there was no significant difference in mean path length in task 1 between 2D and 3D according to the statistical analysis, the score for the 3D system was shorter than that for the 2D system.

We also investigated the average scores of 2D start group and 3D start group with only early phase (from the first to fifth attempts) to work out whether novice trained under a 3D monitor will learn faster than under a 2D monitor or not. The operative time during early phase among 3D group was shorter than that among 2D group only in task 2 [median operative time, 2D vs. 3D (s): task 1, 25.3 vs. 23.7, *P* = 0.350; task 2, 26.3 vs. 23.2, *P* = 0.001; and task 3, 40.7 vs. 39.1, *P* = 0.374]. The path lengths during early phase among 3D group was shorter than that among 2D group in tasks 2 and 3 [median path lengths, 2D vs. 3D (mm): task 1, 1511.6 vs. 1401.5, *P* = 0.068; task 2, 1195.7 vs. 1059.3, *P* < 0.001; and task 3, 2054.9 vs. 1730.8, *P* = 0.008]. The technical errors during early phase among 3D group was shorter than that among 2D group in all tasks [median technical errors, 2D vs. 3D (times): task 1, 1.4 vs. 1.0, *P* = 0.047; task 2, 3.8 vs. 1.1, *P* < 0.001; and task 3, 12.8 vs. 8.6, *P* < 0.001). Each score for all tasks performed under 3D was relatively better compared to those with the 2D system. During early phase, the performance under 3D was significantly better than under 2D for all tasks, especially in technical error.

### Analysis of learning curves

Figure 3B shows the learning curves for the 2D and 3D start groups throughout all attempts. During the first set (first to fifth attempts) of tasks, the 3D start group had shorter operative times and path lengths and fewer errors than the 2D start group. On the other hand, during the later set (sixth to tenth attempts) of tasks, the 3D start group had longer operative times and path lengths and more frequent errors after switching to the 2D system as opposed to performance of the first set.

Table 1 shows the comparison of scores between the first to third attempts and the eighth to tenth attempts at each task to determine whether performance was improved from the early phase to later phases by repetition. Each task 1 score in both groups was significantly improved from the early phase to the later phase of repetition, except that technical errors for the 3D start group in the later phase were similar to those in the early phase. In task 2, all scores in the 2D start group were significantly improved from the early phase to the later phase in contrast to opposite findings for the 3D start group. In the 3D start group, operative times and path lengths were not improved by repetition, and technical errors in the later phase were significantly worse than in the early phase. In task 3, all scores for the 2D start group were significantly improved from the early phase to the later phase, whereas all scores for the 3D start group were similar to those in the early phase.

**Table 1:** Comparison between the first to third attempts and the eighth to tenth attempts

Value	2D start			3D start		
	1st–3rd	8th–10th	*P* value	1st–3rd	8th–10th	*P* value
Operative time (s)						
Task 1	32.1	17.9	<0.001*	28.8	21.6	<0.001*
Task 2	30.7	18.0	<0.001*	25.5	26.5	0.655
Task 3	45.5	33.1	<0.001*	42.2	42.6	0.756
Path length (mm)						
Task 1	1772	1152	<0.001*	1591	1251	<0.001*
Task 2	1492	905	<0.001*	1177	1196	0.715
Task 3	2251	1678	<0.001*	1954	1904	0.351
Technical errors (times)						
Task 1	1.7	0.8	0.015*	1.3	1.3	0.870
Task 2	4.5	1.1	<0.001*	1.4	2.8	0.016**
Task 3	15.6	6.9	<0.001*	9.9	12.3	0.068

* The performance in later phase was significantly better than in early phase

** The performance in later phase was significantly worse than in early phase

Table 2 shows the scores for the fourth to fifth attempts and the sixth to seventh attempts by the 2D start group and 3D start group to reveal whether performance was improved after switching to the opposite monitor.

**Table 2:** Comparison between the fourth to fifth attempts and the sixth to seventh attempts

Value	2D start			3D start		
	4th–5th	6th–7th	*P* value	4th–5th	6th–7th	*P* value
Operative time (s)						
Task 1	24.1	19.5	0.086	21.9	23.7	0.218
Task 2	25.2	18.7	0.001*	21.4	29.0	0.004**
Task 3	38.2	34.8	0.239	38.4	43.5	0.120
Path length (mm)						
Task 1	1419	1257	0.148	1314	1325	0.818
Task 2	1204	970	0.019*	1007	1303	0.003**
Task 3	1905	1725	0.209	1809	1944	0.229
Technical errors (times)						
Task 1	1.6	0.8	0.032*	1.1	1.4	0.231
Task 2	3.1	1.4	0.027*	0.7	3.2	<0.001**
Task 3	11.9	8.0	0.021*	8.2	11.6	0.005**

* The performance in later phase was significantly better than in early phase

** The performance in later phase was significantly worse than in early phase

In task 1, mean operative times and mean path lengths in both groups did not differ significantly after switching to the opposite system. However, technical errors by the 2D start group were significantly improved after switching to the 3D system, whereas the scores for the 3D start group were similar after switching to the 2D system. In task 2, all 2D start group scores were significantly improved just after switching to the 3D system as opposed to the significantly worse scores for the 3D start group after switching to the 2D system. For task 3, mean operative times and mean path lengths in both groups did not differ significantly after switching to the opposite system. However, technical errors by the 2D start group were significantly improved after switching to the 3D system, whereas technical errors were significantly worse in the 3D start group after switching to the 2D system.

## Discussion

Several studies have proposed that stereoscopic vision using 3D results in benefits in laparoscopic performance by trainees [8–12]. The present study also showed that the use of a 3D monitor for training improved laparoscopic surgical performance by novices. Blavier et al. [13] reported a shorter operative time by novices who trained in the 2D environment after switching to a 3D environment and a longer operative time for novices who trained in the 3D environment after switching to a 2D environment. We also showed that the use of a 2D monitor impaired the performance of trainees after training using 3D indicated by a longer operative time due to longer path lengths and more frequent errors. Saseem et al. [14] reported similar findings, especially in complicated tasks. Our results showed that all scores for performance of task 1 (simple task) (median operative time, path length, and technical errors) in the 3D start group did not differ significantly between just before and after switching to a 2D monitor. However, in more complicated tasks, the scores for the 3D start group obviously worsened just after switching monitors, especially the score for technical errors. This inaccurate performance by novices after the loss of depth perception using 3D may be the major reason for the longer operative time under 2D vision.

We considered that there would be differences in the learning effect among trainees depending on their degree of proficiency as surgeons. Therefore, under the same setting, the differences in the learning effect among experienced surgeons might be shorter compared with that among novices. We previously revealed that scores for performance of laparoscopic sutures in the training box by moderately experienced surgeons became significantly worse after switching monitors from 3D to 2D [15]. However, in the task of straight rod transfer through a single loop (which was the same setting as task 2 in this study), the scores for performance by moderately experienced surgeons did not worsen after switching monitors from 3D to 2D. On the other hand, in the present study, the performance of task 2 by novices became significantly worse after switching monitors from 3D to 2D. This suggests that the deleterious effect of loss with depth perception using 3D is dependent not only on the difficulty of the task but also on the lack of experience of the surgeon. When a novice who is trained only using a 3D monitor participates in laparoscopic surgery using 2D for the first time, the surgical team should pay attention to the possibility of more frequent technical errors and longer operative times.

There are several limitations in this study. First, we did not take into consideration the unsuitability of each participant for stereo vision. Several studies about 3D indicated that there are some people who have disadvantages of stereo vision, such as stereo blindness [16, 17], or the side effects (feeling discomfort, headache, or sickness) using a 3D monitor [18]. However, each participant in this study improved their performance and did not feel any discomfort or sickness as side effects by 3D during the trial. We considered that the reason why the participants had no side effects might be stabilizing stereoscope not to move the background displayed on the monitor, or quite short operative time for each subject compared to actual surgery. Second, tasks in this study were simply designed just for novice. Therefore, the statistical difference of scores in each task might not explain the clinical importance by itself and it is unclear whether our performance data from novices using the training box can be extrapolated to actual laparoscopic surgery. However, in actual surgery, there are countless situations that request to perform simple operation such like task 1 and accumulation of each slight difference might become quite a large difference. Therefore, it remains unclear whether novices should start laparoscopic training or experience their first surgery using 3D or 2D. However, considering the obvious better performance of novices using 3D compared with 2D, laparoscopic surgery using 3D is recommended for novices as far as the environment permits. The more frequent errors by novices under 2D compared with 3D in the training box indicate that with poorly experienced surgeons more frequent accidents such as bleeding or injury could occur during actual surgery when switching from 3D to 2D.

In conclusion, for novices, stereoscopic images improved their laparoscopic surgical techniques from the early phase of training. However, it is important to understand the learning effect showing that performance of novices trained only by 3D worsened after changing to the 2D environment even for a simple task.
